# Which surgical technique may yield the best results in large, infected, segmental non-unions of the tibial shaft? A scoping review

**DOI:** 10.1007/s00068-024-02478-y

**Published:** 2024-03-06

**Authors:** Dena Akhoundzadeh, Frank W. Bloemers, Michael H. J. Verhofstad, Linda J. Schoonmade, Leo M. G. Geeraedts

**Affiliations:** 1 Department of Surgery, Section Trauma Surgery, Amsterdam UMC, location VUmc, De Boelelaan 1117, P.O. Box 7057, 1007 Amsterdam, MB Netherlands; 2 Department of Surgery, Section Trauma Surgery, Erasmus MC, Rotterdam, Netherlands; 3 University Library, VU University, Amsterdam UMC, Amsterdam, Netherlands

**Keywords:** Gap nonunion, Tibial diaphysis, Infection, Treatment options, Large bone defects, Scoping review

## Abstract

**Purpose:**

Infected nonunion of the tibia with a large segmental bone defect is a complex and challenging condition for the patient and surgeon. This scoping review was conducted to identify existing evidence and knowledge gaps regarding this clinical scenario. Secondly, the objective of this study was to search for a valid recommendation on the optimal treatment.

**Methods:**

A comprehensive search was conducted in the bibliographic databases: PubMed, Embase.com, and Web of Science Core Collection. Studies reporting on bone transport techniques, the Masquelet technique, and vascularized fibular grafts in bone defects greater than 5 cm were included. Bone healing results and functional results were compared according to duration of nonunion, infection recurrence, bone consolidation, complication rate, external fixation time, and time until full weight-bearing.

**Results:**

Of the 2753 articles retrieved, 37 studies could be included on bone transport techniques (*n* = 23), the Masquelet technique (*n* = 7), and vascularized fibular grafts (*n* = 7). Respective bone union percentages were 94.3%, 89.5%, and 96.5%. The percentages of infection recurrence respectively were 1.6%, 14.4% and 7.0%, followed by respectively 1.58, 0.78, and 0.73 complications per patient.

**Conclusion:**

Bone transport was found to be the most widely studied technique in the literature. Depending on the surgeon’s expertise, vascularized fibular grafts may be held as a favourable alternative. This review indicates that further high-quality research on large bone defects ($\ge$ 5 cm) in patients with infected tibial nonunions is necessary to gain more insight into the potentially beneficial results of vascularized fibular grafts and the Masquelet technique.

**Supplementary Information:**

The online version contains supplementary material available at 10.1007/s00068-024-02478-y.

## Introduction

Infected nonunion of the tibia with a large bone defect is a complex clinical scenario that is often easier to diagnose than to treat [1]. When a tibial shaft fracture fails to heal in 9 months and shows no progressive radiographic healing over 3 consecutive months, it is often defined as a nonunion [2, 3]. Tibial nonunions are common in clinical practice after high energy trauma and are difficult to manage [1–6]. The presence of infection and devascularization in large bone defects prolongs the period of treatment, makes prognosis worse, and in some cases may even lead to amputation of the lower leg [1]. In addition, smoking and diabetes mellitus found to be the most associating patient-depending factors that may contribute to the development or maintenance of a nonunion [7]. Major soft tissue damage, leg-length discrepancy, deformity, and joint stiffness provide functional handicaps and have severe adverse effects on the patient’s quality of life [5, 6, 8]. This challenging and costly management sequentially causes psychological, social, and economic hardships [1, 5, 8].

After diagnosing an infected, segmental nonunion of the tibia, a tailor-made assessment is required to plan an optimally effective treatment program. The infection should be controlled and the nonunion healed [3, 9, 10]. The surgical management has been pioneered by bone transport techniques that use distraction osteogenesis to fill in the large bone gaps [5, 11–18]. After corticotomy, new bone mass is regenerated between the ends of the bone segment. As a result of bifocal or trifocal osteosynthesis, the bone segment unites with the opposite site of the bone defect under compression. The defect size decreases at the same rate as the distraction gap increases [5, 9]. Alternatively, Masquelet developed the induced membrane technique, a two-staged technique consisting of debridement and insertion of antibiotic cement-impregnated spacers to fill the bone gap [19–24]. Bone graft techniques, such as vascularized fibular grafts, may be used as a one-stage or two-stage procedure often combined with external fixation for mechanical stabilization [25–29].

The surgical treatment of infected tibial nonunions with defect sizes *smaller* than 5 cm has extensively been researched. However, little is known about large bone defects ($\ge$ 5 cm). Therefore, the aim of this scoping review was to identify existing evidence and to reveal knowledge gaps regarding this clinical entity. An update of the most common techniques existing for the management of infected nonunions of the tibia with large defect sizes was illustrated: bone transport, Masquelet induced membrane technique, and vascularized fibular grafts. By differentiating between their approaches, an attempt was made to draw a valuable conclusion to which technique yields the best results.

## Methods

This scoping review was reported in accordance with the Preferred Reporting Items for Systematic Reviews and Meta-Analyses Extension for Scoping Reviews (PRISMA-ScR) Statement [30].

### Eligibility criteria

Studies were included if they met the predetermined criteria: (i) nonunions; (ii) infection; (iii) segmental bone defects of more than 5 cm; (iv) located in tibial diaphysis/shaft; (v) treated with surgical techniques addressing bone transport, vascularized fibular grafts, and the Masquelet induced membrane technique; (vi) randomized controlled trials, case control studies and pro-retrospective studies, and case series of two or more cases. The search was limited to English language and human studies only. No limits were placed on year of publication. Studies were excluded if they met the predetermined criteria: (i) systematic reviews, meta-analyses, case reports of less than two cases, editorials, (ii) other language than English, (iii) studies reporting on animals or children (< 18 years), (iv) nonsegmental fractures, (v) aseptic fractures, and (vi) locations other than the tibial diaphysis/shaft. Duplicates were excluded.

### Search methods

A comprehensive search was performed in the bibliographic databases PubMed, Embase, and Web of Science Core collection from inception up to June 5, 2023, in collaboration with a medical librarian (LJS). Search terms included controlled terms (MesH in PubMed and Emtree in Embase) as well as free text terms. The following terms were used (including synonyms and closely related words) as index terms or free-text words: ‘tibial fractures’ and ‘non-union infections’ and ‘surgical treatment’. The search was performed without language or date restrictions. The full search strategies for all databases can be found in the Supplementary Information shown in Appendix A. Duplicate articles were excluded (LJS) using Endnote X20.01 (Clarivate^tm^), following the Amsterdam Efficient Deduplication (AED) method [31]  and the Bramer method [32].

### Selection process

Two reviewers (DA and LMGG) independently screened all potentially relevant titles and abstracts for eligibility with the use of Rayyan QCRI [33], a free online web tool for systematic reviews.

**Fig. 1:**
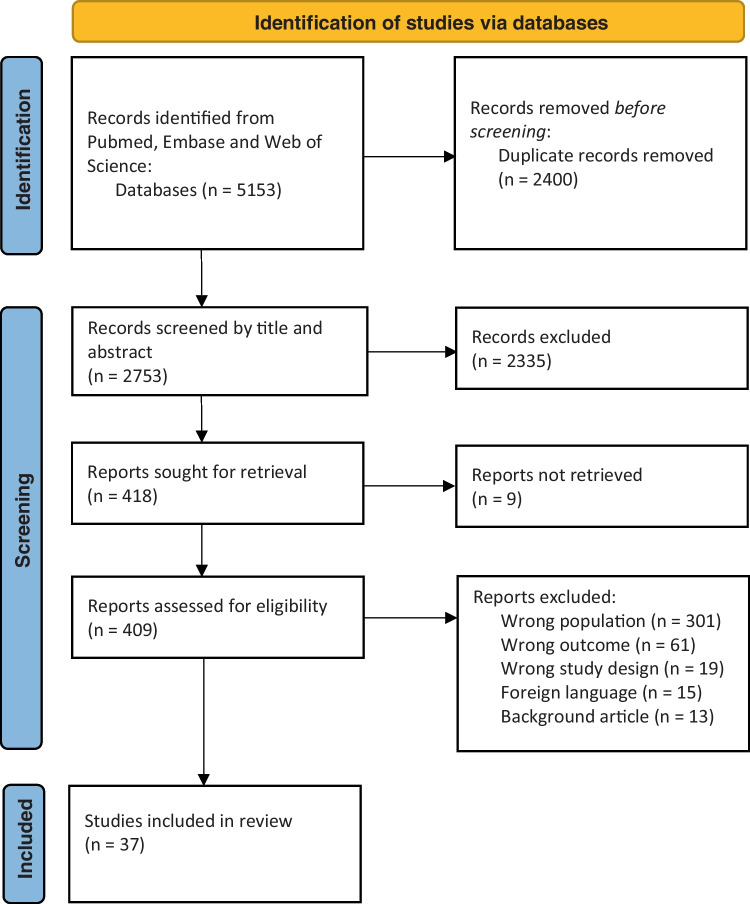
Study selection process of the scoping review, according to PRISMA guidelines [34]

Full-text papers were ordered for those studies which met the eligibility criteria. Two reviewers (DA and LMGG) independently reviewed each full-text paper against the eligibility criteria and included pertinent studies in the scoping review. Disagreements between the reviewers in respect to the study eligibility were resolved with discussion between the two reviewers until a consensus was reached. The overall search strategy, the selection process, and the results of the search are presented in the PRISMA flowchart illustrated in Fig. 1.

### Data collection

From the selected studies, the following data were extracted: name of author and publication year, study design, number of patients, percentage of male and female, mean age in years, mean size of bone defects (cm), mean size of skin defects (cm^2^), and the number of previous operations per patient. The following healing results and functional results were extracted from studies: applied technique, method, mean follow-up time (months), union time (months), percentage of infection recurrence, percentage of complications, percentage of bone union, external fixation time (months), and the time until full weight-bearing (months).

### Statistical analysis

All data were documented and analysed in the statistical analysis software SPSS, version 22. Normally distributed data were presented as mean and standard deviation, not normally distributed data were presented as median and interquartile range (IQR), and categorical data were presented as absolute and percentage. Missing data were excluded from statistical analysis according to pairwise deletion.

## Results

Table 1 shows the results of the literature search in PubMed, Embase, and Web of Science. The literature search generated a total of 5153 references: 1612 in PubMed, 2121 in Embase, and 1420 in Web of Science Core Collection. After removing duplicates, 2753 references, published from 1963 to 2023, remained. The flow chart of the search process is presented in Fig. 1.

**Table 1:** Results obtained in PubMed, Embase, and Web of Science on the 5th of June 2023

Database	Results
Pubmed	1612
Embase	2121
Web of Science	1420
Total	5153
After removing duplicates	2753

After screening by titles and abstracts, a total of 418 studies remained. Ultimately, 37 studies met the inclusion criteria after review of full-text articles (Fig. 1). Of the 37 studies, 28 were retrospective case series, 5 were retrospective comparative studies, and 4 were prospective cohort studies. Main study and patient characteristics and an overview of the studies are shown in Table B1 and can be found in Appendix B. The interventions and outcomes extracted from the studies are shown in Table B2 (Appendix B).

### Patient characteristics, interventions, and outcomes

A total of 685 patients with infected, segmental nonunions of the tibial shaft with a minimum defect size of 5 cm were included. A total of 523 patients were treated by bone transport techniques of which 308 were treated by the Ilizarov method, 87 patients with a monolateral rail fixator system, and 40 patients with acute shortening and lengthening using variable frames. The Ilizarov method was used in combination with an antibiotic cement spacer in 81 patients, teriparatide injection in 20 patients, and the Taylor spatial frame in 10 patients. A total of 105 patients were treated by the Masquelet induced membrane technique. A total of 57 patients were treated by the vascularized fibular graft technique. An overview of the patient characteristics of each technique is listed in Table 2.

**Table 2:** Characteristics of patients with infected, segmental nonunions of the tibial shaft treated by bone transport, Masquelet, and vascularized fibular graft

Variable	Bone transport *n* = 523	Masquelet *n* = 105	Vascularized fibular graft *n* = 57
Percentage male (%)	82.6	86.3	83.9
Mean age (years)	35.9 (IQR 24.10)*	45.3 (SD 7.68) Median: 48.5 (IQR 11.10)	39.0 (SD 7.32) Median: 37.4 (IQR 9.50)
Defect size (cm)	8.0 (IQR 2.90)*	6.7 (SD 1.13) Median: 6.5 (IQR 1.95)	10.3 (SD 2.61) Median: 9.6 (IQR 3.95)
Mean number of previous operations	3.7 (SD 2.15)	2.8 (SD 1.41)	5.1 (SD 2.73)

Absolute amount, percentages, or median and interquartile range

The data did not be reported in studies

*SD* standard deviation

*IQR* interquartile range

^1^Population too small for IQR/SD

^*^Non-normally distributed data

### Bone transport

The interventions in the studies using the Ilizarov method as treatment for infected, segmental nonunion of the tibia mainly included three parts: radical debridement and resection of necrotic bone and soft tissue; bone transportation using the Ilizarov circular frame, compression-distraction osteosynthesis; and eventually the administration of systemic antibiotics [35–52]. In 81 patients, this technique was combined with an antibiotic cement spacer, whereas 20 patients were given a teriparatide injection. A common variation of bone transport used in a total of 87 patients in this scoping review is the monolateral rail fixator. In contrast to the circular frame, this rail system is fastened on the unilateral side of the tibia. Alternatively, the acute shortening and lengthening technique was used in 40 patients. This technique involved a one-stage treatment by acute compression of the defect area and lengthening from the healthy part of the bone through another osteotomy. The Taylor spatial frame, which was used in a total of 10 patients, is a further development of the classic Ilizarov technique. The frame is also secured in the bone with thin pins and screws. The main difference is the connection between the two rings, which are connected to each other via six ‘struts’.

### Masquelet

The interventions of the studies reporting on the Masquelet technique as treatment for infected, segmental nonunion of the tibial shaft included a two-staged surgical procedure. The first stage consisted of filling the bone gap with a polymethyl methacrylate (PMMA) cement spacer. Secondly, free vascularized soft tissue transfers and conventional bone grafting were used to fill in the bone defect surrounded by the cement induced membrane [53–57].

### Vascularized fibular graft

The interventions in the studies using vascularized fibular grafts were radical debridement and resection of necrotic bone and soft tissue, osteocutaneous free fibular vascularized bone transfers with a large skin island, and eventually the application of external fixators [58–62].

Comparison of the outcomes of the three different techniques can be found in Table 3. Further aspects were listed in Table 2B and can be found in Appendix B.

**Table 3:** Outcomes of patients with infected, segmental nonunions of the tibial shaft treated by bone transport, Masquelet, and vascularized fibular graft

Variable	Bone transport *n* = 523	Masquelet *n* = 105	Vascularized fibular graft *n* = 57
Follow-up time (months)	29.4 (IQR 12.83)*	29.5 (SD 11.34) Median: 32,9^1^	34.6 (SD 8.37) Median: 34.5 (IQR 16.55)
Mean union time (months)	10.0 (SD 5.58)	8.6 (SD 1.81)	6.8 (SD 2.51)
Infection recurrence (%)	1.6	14.4	7.0
Bone union (%)	94.3	89.5	96.5
External fixation time (months)	11.5 (SD 3.72)	7.3^1^	9.7^1^
Time until full weight-bearing (months)	8.8 (SD 1.98)	7.6 (SD 0.53)	12.1^1^
Mean number of complications per patient	1.58 (SD 0.89)	0.78 (SD 0.37)	0.73 (SD 0.75)

Absolute amount, percentages, or median and interquartile range

The data did not be reported in studies

*SD* standard deviation

*IQR* interquartile range

^1^Population too small for IQR/SD

^*^Non-normally distributed data

### Complications

Pin-track infections (*n* = 212, 42.2%) counted the most common complications reported in the studies of bone transport, followed by joint stiffness (*n* = 112, 22.4%) and axial deformity (*n* = 79, 15.8%). These complications were not reported in the studies of the Masquelet technique and vascularized fibular grafts. The most common complication reported in studies of the Masquelet technique was hematoma (*n* = 3, 12.0%) and in vascularized fibular grafts: stress fractures (*n* = 7, 12.3%). Furthermore, thrombosis was reported in one patient of the Masquelet technique (*n* = 1, 4.0%) and one patient of vascularized fibular grafts (*n* = 1, 1.8%). Thrombosis and hematoma are not reported in studies of bone transport. An overview of reported complications is listed in Table 4.

**Table 4:** List of reported complications in studies reporting on bone transport, Masquelet, and vascularized fibular graft

Complications	Bone transport *n* (%)	Masquelet *n* (%)	Vascularized fibular graft *n* (%)
Pin-track infection	212 (42.2)		1 (1.8)
Joint stiffness	112 (22.4)	10 (9.5)	1 (1.8)
Infection recurrence	31 (6.2)	8 (7.6)	4 (7.0)
Axial deformity	79 (15.8)		2 (3.5)
Loosening of pins	11 (2.2)		
Breakage of pins	8 (1.6)		
Malunion	34 (6.8)	2 (8.0)	1 (1.8)
Refracture	7 (1.4)	2 (1.9)	
Limb length discrepancy	46 (9.2)	3 (2.9)	
Limb edema	11 (2.2)		
Neurovascular injury	37 (7.4)		
Thrombosis		1 (4.0)	1 (1.8)
Hematoma		3 (12.0)	4 (7.0)
Pseudoarthrosis	1 (0.2)		
Amputation	1 (0.2)	1 (4.0)	2 (3.5)
Stress fracture	3 (0.6)		7 (12.3)

The percentage of each complication was calculated for each group separately

## Discussion

This scoping review was conducted to evaluate three common surgical techniques in the treatment of patients with an infected, segmental nonunion of the tibial shaft with large ($\ge$ 5 cm) bone defects. This study was initially set up as a systematic review. However, given the heterogeneity and the inability to perform a meta-analysis, designed as a scoping review to summarize the existing literature reporting on radiographic and functional outcomes: union rates, percentages of infection recurrence, bone union duration, and complication rates. Furthermore, this scoping review recommends the Delphi survey technique as a useful method of choice for studies in future perspective to help enhance effective decision-making in the treatment of this clinical entity [63].

Vascularized fibular grafts showed the highest percentage of bone union, the shortest duration of nonunion, and a relatively low infection recurrence percentage compared to Masquelet and bone transport techniques. These results are in accordance with studies indicating that vascularized fibular grafts are favourable in infected nonunions of the tibia with large ($\ge$ 5 cm) bone defects [64–66]. The bone gap is filled in with large bone grafts all together with the recipient vessel away from the injured zone to provide microvascular anastomosis. The fibula is a popular bone for transplantation, because it is easy to align, has great strength, and can bridge large gaps. A unique challenge is providing osteogenic cells that could participate in the healing process and can respond to changes in functional loading by adaptive remodelling and hypertrophy [64–66]. Therefore, an additional challenge is the availability of surgeons who master this technique.

Bone transport was found to be the most widely used technique in the literature. This technique has a high union rate and low rates of persistent infection compared to the Masquelet technique and vascularized fibular grafts. In addition, full weight-bearing is possible immediately after application of the distraction device [13]. Commonly reported complications such as joint stiffness, axial deformity, and pin-track infections are less likely to occur in the Masquelet technique and vascularized fibular grafts. Hematoma and thrombosis are merely reported in studies of Masquelet. This finding is contrary to previous studies which have suggested that circulatory problems are classic complications that often come with vascularized fibular grafts [67]. In studies of vascularized fibular grafts, stress fractures were the most frequently reported. The result of this characteristic complication agrees with those obtained in the study of Kadhim et al. [67].

The external fixation system of bone transport is mainly used by the methods of Ilizarov. Rohilla et al. [68] showed a lower complication rate, shorter duration of bone union, and overall higher ASAMI bone and function scores when replacing Ilizarov’s circular frame by a rail fixator system. Tetsworth et al. [5] demonstrated a lower rate of complications and a slightly better radiographic outcome when comparing bone transport to acute shortening and lengthening. The comparative study of Yushan et al. [69] found a lower complication rate and higher ASAMI-scores when using trifocal compression compared to bifocal compression in the methods of Ilizarov. Comparing to other bone transport techniques, the study of Gupta et al. [70] showed a shorter duration of nonunion, lowest persistent infection rate, and the highest union rate when using the monolateral rail fixator system. As an alternative to the established Ilizarov circular frame, three studies used the Ilizarov method in combination with antibiotic cement spacers. This two-staged procedure showed lower percentages of infection recurrence than compared to the Ilizarov method alone.

The induced membrane technique as reported firstly by Masquelet [19] does not regenerate bone but relies on a two-staged procedure. The first stage consists of radical resection of all necrotic and infected tissue, stable internal or external fixation of the defect, and well-vascularized soft tissue coverage. An antibiotic-loaded PMMA-block is inserted in the bony gap for a number of weeks. The inflammatory reaction to this foreign body results in the development of a well-vascularized membrane with high cellular and humoral content. The second stage consists of enucleating the cement spacer and filling the cavity with autologous bone grafts [26, 71]. The Masquelet induced membrane technique is a simple technique and has the advantage that the infection can subside during the time of the cement block in the tibial bone gap. By way of contrast, the chief drawback of this technique is the intensive and prolonged standardized treatment. Moreover, the need for large amounts of bone graft could result in donor site morbidity [5, 19].

### Limitations

There are several limitations in this review. Firstly, data were extracted from mostly retrospective observational studies and only four prospective studies. Confounders may be less reliable and suffer from both information and selection bias. The second main limitation is the small sample sizes of studies reporting on the Masquelet induced membrane technique and the vascularized fibular graft technique compared to studies of bone transport. Due to a low incidence of patients with infected, nonunion of the tibia with a large ($\ge$ 5 cm) defect size, studies included in this review contained a heterogeneous group of patients. Also, only English articles were included in this review. Relevant articles could have been missed based on language criteria. Ideally, clinical trials including large numbers of patients are needed to carefully formulate a recommendation based on the three techniques discussed in the current review. Future studies that focus on large defect sizes (≥ 5 cm) in the treatment of infected nonunions of the tibial shaft are needed and should be prospective. Due to the low incidence of large defects, such work is only possible in a multicentric fashion. Even then, randomization between treatment options will be difficult, if not impossible to realize. Improvement of scientific evidence will presumably come from prospective registries.

## Conclusion

This scoping review recommends vascularized fibular grafts based on the highest percentage of bone union, the shortest duration of nonunion, and a relatively low infection recurrence percentage. Nonetheless, due to the variety in group size and heterogeneity of the studies, the results of this review need to be interpreted with great caution. Furthermore, future prospective studies that focus on large defect sizes ($\ge$ 5 cm) in the treatment of infected nonunions of the tibial shaft are needed.

## Supplementary Information

Supplementary file1 (DOCX 58.8 KB)

## Data Availability

All data supporting the findings of this study are available within the paper and its Supplementary Information. Data are provided in Supplementary Information (Appendix A) and Supplementary Tables (Appendix B), Table B1 & Table B2.
