# Supplementary material for: Which surgical technique may yield the best results in large, infected, segmental non-unions of the tibial shaft? A scoping review
Source: Eur J Trauma Emerg Surg. 2024 Mar 6;50(4):1537–45. doi: 10.1007/s00068-024-02478-y (PMC11458670; doi:10.1007/s00068-024-02478-y)
Supplement: Supplementary file 1 — Supplementary file1 (DOCX 58.8 KB) [file 68_2024_2478_MOESM1_ESM.docx]

Appendix A – Supplementary Information

# Search Histories June 5, 2023

### PubMed History June 5, 2023

| **Search** | **PubMed Query – June 5, 2023** | **Items found** |
| --- | --- | --- |
| #4 | #1 AND #2 AND #3 | 1,612 |
| #3 | "External Fixators"[Mesh] OR "Bone Transplantation"[Mesh] OR "Ilizarov Technique"[Mesh] OR "Orthopedic Procedures"[Mesh] OR “external fixat*”[tiab] OR Masquelet*[tiab] OR Ilizarov[tiab] OR “rail fixat*”[tiab] OR “fibular graft*”[tiab] OR “bone graft*”[tiab] OR “bone transport*”[tiab] OR “bone transfer*”[tiab] OR “free vasculari*”[tiab] OR non-vasculari*[tiab] OR “ring fixat*”[tiab] OR “induced membrane”[tiab] OR reconstructive[tiab] OR reconstruction[tiab] OR surgery[tiab] OR surgeries[tiab] OR surgical[tiab] OR operation*[tiab] OR operative[tiab] OR technique*[tiab] OR management*[tiab] | 5,413,119 |
| #2 | ("Infections"[Mesh] OR infect*[tiab] OR septic[tiab] OR osteomyelitis[tiab] OR osteitis[tiab]) AND (nonunion[tiab] OR non-union[tiab] OR “segmental defect*”[tiab] OR “bone loss*”[tiab]) | 8,514 |
| #1 | "Tibial Fractures"[Mesh] OR ("Tibia"[Mesh] OR tibia*[tiab]) AND ("Fractures, Bone"[Mesh] OR fracture*[tiab]) | 28,972 |

###

### Embase.com History June 5, 2023

| **Search** | **Embase.com Query – June 5, 2023** | **Items found** |
| --- | --- | --- |
| #4 | #1 AND #2 AND #3 | 2,121 |
| #3 | 'external fixator'/exp OR 'bone transplantation'/exp OR 'Ilizarov external fixator'/exp OR 'orthopedic surgery'/exp OR 'reconstructive surgery'/exp OR ‘external fixat*’:ab,ti,kw OR Masquelet*:ab,ti,kw OR Ilizarov:ab,ti,kw OR ’rail fixat*’:ab,ti,kw OR ‘fibular graft*’:ab,ti,kw OR ‘bone graft*’:ab,ti,kw OR ‘bone transport*’:ab,ti,kw OR ‘bone transfer*’:ab,ti,kw OR ‘free vasculari*’:ab,ti,kw OR non-vasculari*:ab,ti,kw OR ‘ring fixat*’:ab,ti,kw OR ‘induced membrane’:ab,ti,kw OR reconstructive:ab,ti,kw OR reconstruction:ab,ti,kw OR surgery:ab,ti,kw OR surgeries:ab,ti,kw OR surgical:ab,ti,kw OR operation*:ab,ti,kw OR operative:ab,ti,kw OR technique*:ab,ti,kw OR management:ab,ti,kw | 7,150,723 |
| #2 | ('infection'/exp OR 'osteomyelitis'/exp OR infect*:ab,ti,kw OR septic:ab,ti,kw OR osteomyelitis:ab,ti,kw OR osteitis:ab,ti,kw) AND (nonunion:ab,ti,kw OR non-union:ab,ti,kw OR ‘segmental defect’:ab,ti,kw OR ‘bone loss*’:ab,ti,kw) | 11,061 |
| #1 | 'tibia fracture'/exp OR ('tibia'/exp OR tibia*:ab,ti,kw) AND ('fracture'/exp OR fracture*:ab,ti,kw) | 38,939 |

###

### Web of Science Core Collection History June 5, 2023

| **Search** | **Web of Science Core Collection Query – June 5, 2023** | **Items found** |
| --- | --- | --- |
| #4 | #1 AND #2 AND #3 | 1,420 |
| #3 | TS = (“external fixat*” OR “bone transplantation” OR “Ilizarov external fixator” OR “orthopedic surgery” OR “reconstructive surgery” OR Masquelet* OR Ilizarov OR “rail fixat*” OR “fibular graft*” OR “bone graft*” OR “bone transport*” OR “bone transfer*” OR “free vasculari*” OR non-vasculari* OR “ring fixat*” OR “induced membrane” OR reconstructive OR reconstruction OR surgery OR surgeries OR surgical OR operation* OR operative OR technique* OR management) | 9,105,973 |
| #2 | TS = ((infect* OR septic OR osteomyelitis OR osteitis) AND (nonunion OR non-union OR “segmental defect” OR “bone loss*”)) | 7,795 |
| #1 | TS= (“tibia fracture” OR (tibia* AND fracture*)) | 22,717 |

Appendix B – Supplementary Tables

**Table B1**. Study and patient characteristics of patients with infected, segmental nonunions of the tibial shaft.

| Article No.  [ref] | Name author (year of publication) | Study design | Number of patients | Male (%) | Female (%) | Mean age  (years) | Mean size bone defects  (cm) | Mean size skin defects  (cm^2^) | | Mean previous operations (per patient) |
| --- | --- | --- | --- | --- | --- | --- | --- | --- | --- | --- |
| 1. | **Minami et al. (1992)** | Retrospective case series | 8 | 87.5 | 12.5 | 36.8 | 8.9 | | __ | __ |
| 2. | **Dendrinos et al. (1995)** | Retrospective case series | 28 | 82.0 | 18.0 | 37.0 | 6.0 | | __ | 4.0 |
| 3. | **Doi et al. (1995)** | Retrospective case series | 14 | 85.7 | 14.3 | 46.3 | 9.1 | | 68.1 | 3.0 |
| 4. | **Hoogendoorn et al. (2003)** | Retrospective case series | 4 | 75.0 | 25.0 | 41.3 | 14.8 | | __ | 3.5 |
| 5. | **Yajima et al. (2004)** | Retrospective case series | 7 | 85.7 | 14.3 | 48.1 | 11.7 | | __ | 4.7 |
| 6. | **Fabry et al. (2005)** | Retrospective case series | 7 | 71.4 | 28.6 | 30.9 | 8.9 | | __ | __ |
| 7. | **Schöttle et al. (2005)** | Retrospective case series | 6 | 83.3 | 16.7 | 49.0 | 6.5 | | 80.0 | 2.3 |
| 8. | **Magadum et al. (2006)** | Retrospective case series | 27 | 100.0 | 0.0 | 39.0 | 10.0 | | __ | 2.0 |
| 9. | **Sen et al. (2006)** | Retrospective case series | 8 | 100.0 | 0.0 | 39.4 | 6.4 | | __ | 3.6 |
| 10. | **Bumbasirevic et al. (2009)** | Retrospective case series | 30 | 96.6 | 3.3 | 30.4 | 5.7 | | __ | 1.3 |
| 11. | **Sun et al. (2009)** | Retrospective case series | 5 | 80.0 | 20.0 | 26.0 | 7.4 | | __ | __ |
| 12. | **Megas et al. (2010)** | Retrospective case series | 4 | 75.0 | 25 | 29.8 | 8.3 | | __ | 5.3 |
| 13. | **Sala et al. (2011)** | Retrospective comparative case series | 10 | 70.0 | 30.0 | 35.7 | 8.8 | | __ | 4.7 |
| 14. | **Xu et al. (2013)** | Retrospective case series | 25 | 80.0 | 20.0 | 33.7 | 7.1 | | 6.7 | 8.8 |
| 15. | **Peng et al. (2015)** | Retrospective case series | 58 | 65.5 | 34.5 | 29.4 | __ | | __ | 6.3 |
| 16. | **Aktuglu et al. (2016)** | Retrospective case series | 6 | 100.0 | 0.0 | 31.8 | 9.3 | | __ | __ |
| 17. | **Eralp et al. (2016)** | Retrospective comparative study | 13  32 | __ | __ | 38.9  34.2 | 9.4  6.8 | | __ | __ |
| 18. | **Gupta et al. (2016)** | Prospective cohort study | 6 | 83.3 | 16.7 | 34.2 | 5.9 | | __ | 2.3 |
| 19. | **Rohilla et al. (2016)** | Prospective cohort study | 35 | 85.7 | 14.3 | 36.1 | 7.3 | | 12.5 | 1.2 |
| 20. | **El-Alfy (2017)** | Retrospective case series | 28 | 82.1 | 17.9 | 37.0 | 8.0 | | 35.0 | __ |
| 21. | **Zhang et al. (2017)** | Retrospective case series | 16 | 56.3 | 43.7 | 39.1 | 10.9 | | 40.4 | __ |
| 22. | **Gupta et al. (2018)** | Retrospective case series | 14 | 92.9 | 17.1 | 38.1 | 6.4 | | __ | __ |
| 23. | **Siboni et al. (2018)** | Retrospective case series | 8 | 87.5 | 12.5 | 55.1 | 8.3 | | __ | __ |
| 24. | **Kapukaya et al. (2020)** | Retrospective case series | 19 | 73.7 | 26.3 | 37.4 | __ | | __ | 9.0 |
| 25. | **Kushwaha et al. (2020)** | Prospective cohort study | 21 | 90.5 | 9.5 | 29.4 | __ | | __ | __ |
| 26. | **Lu et al. (2020)** | Retrospective case series | 2 | 50.0 | 50.0 | 53.5 | 5.9 | | __ | 2.0 |
| 27. | **Wadhwani et al. (2020)** | Retrospective case series | 15 | 93.3 | 6.7 | 29.7 | 7.6 | | __ | __ |
| 28. | **Yushan et al. (2020)** | Retrospective comparative study | 37 | 75.7 | 24.3 | 40.1 | 10.3 | | __ | 2.8 |
| 29. | **Kinik et al. (2021)** | Retrospective case series | 30 | 93.3 | 6.7 | 39.5 | 8.1 | | __ | 2.9 |
| 30. | **Ma et al. (2021)** | Retrospective case series | 3 | 100.0 | 0.0 | 37.0 | 10.0 | | __ | __ |
| 31. | **Miraj et al. (2021)** | Retrospective case series | 11 | 90.9 | 9.1 | 34.1 | 14.4 | | __ | __ |
| 32. | **Pesciallo et al. (2021)** | Retrospective case series | 3 | 100.0 | 0.0 | 47.7 | 5.3 | | __ | __ |
| 33. | **Rollo et al. (2021)** | Retrospective comparative study | Norm: 20  Teri: 20 | 90.0  90.0 | 10.0  10.0 | __ | Norm: 9.2  Teri: 9.1 | | __ | __ |
| 34. | **Van Vught et al. (2021)** | Retrospective case series | 2 | 100.0 | 0.0 | 48.5 | 6.8 | | __ | __ |
| 35. | **Khaled et al. (2022)** | Prospective single-center study | 23 | 82.6 | 17.4 | 24.7 | 6.7 | | __ | 2.8 |
| 36. | **Garabano et al. (2022)** | Retrospective case series | 17 | 70.6 | 29.4 | 45.9 | 7.0 | | __ | 2.6 |
| 37. | **Zhang et al. (2023)** | Retrospective comparative study | 63 | 79.4 | 20.6 | 36.9 | 6.9 | | 17.5 | 5.2 |

__ The data did not be reported in studies.

**Table B2**. Intervention and outcomes of patients with infected, segmental nonunions of the tibial shaft.

| Article No. | Method | Mean follow-up time (months) | Union time (months) | Infection recurrence (%) | Complications (%)  (per patient) | Bone union  (%) | External fixation time  (months) | Time until full weight-bearing (months) |
| --- | --- | --- | --- | --- | --- | --- | --- | --- |
|  |  | **Bone transport** |  |  |  |  |  |  |
| 2. | Ilizarov | 39.0 | 6.0 | 1/28  (3.6%) | 25/28  (89.2%)  2.54(71/28) | 24/28  (85.7%) | __ | Immediately after application of circular frame |
| 4. | Ilizarov | __ | 21.7 | 0/3  (0.0%) | 3/3  (100.0%)  2.67(8/3) | 3/3  (100.0%) | __ | __ |
| 6. | Ilizarov | 6.6 | __ | 0/7  (0.0%) | 1/7  (14.2%)  0.14(1/7) | 7/7  (100.0%) | 16.9 | __ |
| 8. | Ilizarov | 27.0 | 6.3 | 1/27  (3.7%) | __  1.11(30/27) | 26/27  (96.3%) | __ | 10.2 |
| 9. | Acute shortening and lengthening | 32.5 | 11.4 | 0/8  (0.0%) | __ | 8/8  (100.0%) | 10.4 | __ |
| 10. | Ilizarov | 99.0 | 4.5 | 0/30  (0.0%) | __  1.40(42/30) | 29/30  (97.0%) | 9.7 | During bony maturation |
| 12. | Ilizarov | __ | __ | 0/4  (0.0%) | 4/4  (100.0%)  2.00(8/4) | 4/4  (100.0%) | 9.4 | __ |
| 13. | Ilizarov/TSF | __ | 3.9 | 0/10  (0.0%) | 10/10  (100.0%)  2.4(24/10) | 10/10  (100.0%) | 14.3 | __ |
| 14. | Ilizarov | 27.6 | 8.0 | 0/25  (0.0%) | 6/25  (24.0%)  0.28(7/25) | 25/25  (100.0%) | 10.1 | __ |
| 15. | Ilizarov combined with antibiotic cement spacer | 31.6 | 10.6 | 1/58  (1.7%) | __  0.67(39/58) | 58/58  (100.0%) | __ | __ |
| 16. | Ilizarov | __ | 10.4 | 0/6  (0.0%) | 5/6  1.0(6/6) | 6/6  (100.0%) | 13.9 | Immediately after application frame |
| 17. | Ilizarov vs. acute shortening and lengthening | 39.2  39.9 | __ | 0/13  (0.0%)  0/32  (0.0%) | __ | 13/13  (100.0%)  32/32  (100.0%) | __ | __ |
| 19. | Ilizarov | 25.4 | __ | 0/35  (0.0%) | __  2.40(84/35) | 32/35  (91.4%) | 11.9 | __ |
| 20. | Ilizarov | 39.0 | __ | 0/28  (0.0%) | __  0.93(26/28) | 13/28  (46.4%) | 13.0 | __ |
| 21. | Ilizarov | 29.5 | 8.2 | 0/16  (0.0%) | __  2.06(33/16) | 14/16  (87.5%) | 12.0 | __ |
| 22. | Monolateral fix. ex. system | 24.0 | 22.2 | 0/14  (0.0%) | __  0.50(7/14) | 14/14  (100.0%) | 11.2 | __ |
| 25. | Monolateral fix. ex. system | 29.5 | 11.0 | 5/21  (23.8%) | 4/21  (19.0%)  __ | 21/21  (100.0%) | 11.2 | Immediately after removal of fix. ex. |
| 26. | Ilizarov | 21.0 | 8.9 | 0/2  (0.0%) | __ | 2/2  (100.0%) | 3.5 | __ |
| 27. | Monolateral rail fix. system | 37.5 | __ | 0/15  (0.0%) | __  2.27(34/15) | 11/15  (73.3%) | 12.2 | __ |
| 28. | Monolateral rail fix. system | 29.5 | BF group:  16.9  TF group:  11.5 | BF group:  0/21  (0.0%)  TF group:  0/16  (0.0%) | BF group:  __  3.10(65.1/21)  TF group:  __  2.80(44.8/16) | BF group:  21/21  (100.0%)  TF group:  16/16  (100.0%) | BF group:  5.2  TF group:  2.7 | __ |
| 29. | Ilizarov | 32.5 | __ | 0/30  (0.0%) | 30/30  (100.0%)  1.97(59/30) | 30/30  (100.0%) | 13.7 | __ |
| 31. | Ilizarov | __ | 10.0 | 0/11  (0.0%) | __ | 11/11  (100.0%) | __ | __ |
| 33. | Ilizarov (Norm) vs.  Ilizarov combined with teriparatide injection (Teri) | Norm: 20.4  Teri:  19.2 | Norm:  2.6  Teri:  2.6 | Norm: 0/20  (0.0%)  Teri:  0/20  (0.0%) | Norm:  __  1.10(22/20)  Teri:  __  1.10 (22/20) | Norm: 20/20  (100.0%)  Teri:  20/20  (100.0%) | Norm:  19.2  Teri:  16.2 | __ |
| 35. | Masquelet-Ilizarov | 26.4 | __ | 0/23  (0.0%) | __ | 23/23  (100%) | __ | 7.4 |
|  |  | **Masquelet** |  |  |  |  |  |  |
| 7. | Masquelet | 36.0 | 8.5 | 0/6  (0.0%) | __  1.16(7/6) | 5/6  (83.3%) | 7.3 | 8.0 |
| 18. | Masquelet | 21.5 | 10.5 | 0/6  (0.0%) | 1/6  (16.7%)  __ | 5/6  (83.3%) | __ | 7.0 |
| 23. | Masquelet | __ | __ | 4/8  (50.0%) | 3/8  (37.5%)  0.75(6/8) | 7/8  (87.5%) | __ | __ |
| 32. | Masquelet | __ | 7.2 | 0/3  (0.0%) | __ | 3/3  (100.0%) | __ | __ |
| 34. | Masquelet | 14.5 | 11.0 | 0/2  (0.0%) | __ | 2/2  (100.0%) | __ | __ |
| 36. | Masquelet | 42.6 | 7.8 | 1/17  (5.9%) | __ | 16/17  (94.1%) | __ | __ |
| 37. | Masquelet | 32.9 | 6.5 | 4/63  (6.3%) | 19/63  (30.2%)  0.42(27/63) | 63/63  (100%) | __ | 7.8 |
|  |  | **Vascularized fibular graft** |  |  |  |  |  |  |
| 1. | Vascularized fibular graft | 44.6 | 6.5 | 0/8  (0.0%) | 1/8  (12.5%)  __ | 8/8  (100.0%) | __ | __ |
| 3. | Vascularized fibular graft | 34.5 | 4.9 | 0/14  (0.0%) | 3/14  (21.4%)  0.29(4/14) | 13/14  (92.9%) | __ | 14.7 |
| 4. | Vascularized fibular graft | __ | 12.0 | 0/1  (0.0%) | 1/1  (100.0%)  2.0(2/1) | 1/1  (100.0%) | __ | __ |
| 5. | Vascularized fibular graft | __ | 6.3 | 3/7  (42.9%) | 3/7  (42.9%)  0.57(4/7) | 6/7  (85.7%) | __ | __ |
| 11. | Vascularized fibular graft | 25.8 | 4.5 | 0/5  (0.0%) | __ | 5/5  (100.0%) | __ | 9.6 |
| 24. | Vascularized fibular graft | 27.0 | 7.4 | 1/19  (5.3%) | __  0.11(2/19) | 19/19  (100.0%) | __ | __ |
| 30. | Vascularized fibular graft | 41.3 | 5.7 | 0/3  (0.0%) | 1/3  (33.3%)  0.67(2/3) | 3/3  (100.0%) | 9.7 | __ |

__ The data did not be reported in studies.

BF bifocal, TF trifocal, TSF Taylor Spatial Frame, fix. fixation, ex. external, vs. versus
